# Supplementary figures and images for: Bacterially produced metabolites protect C. elegans neurons from degeneration
Source: PLoS Biol. 2020 Mar 24;18(3):e3000638. doi: 10.1371/journal.pbio.3000638 (PMC7092960; doi:10.1371/journal.pbio.3000638)

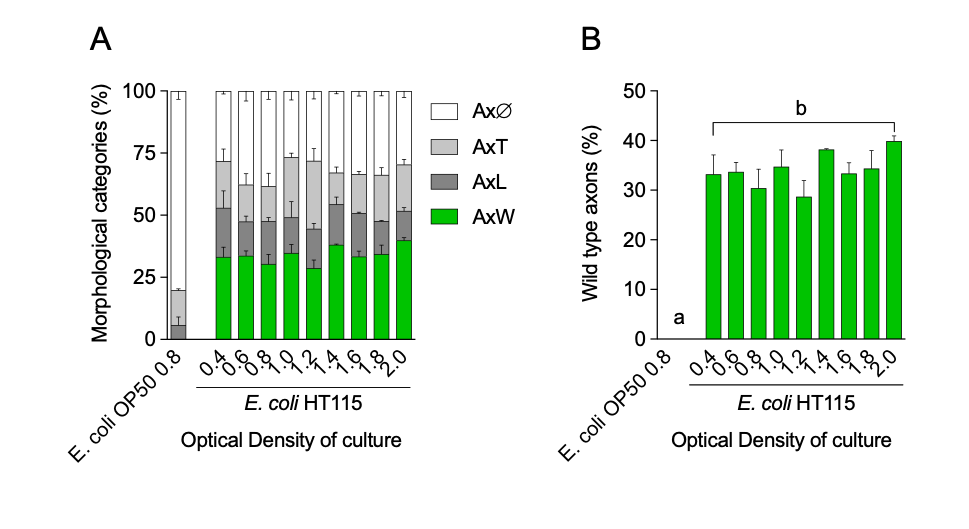

Supplement: S1 Fig — (A and B) All axonal categories (A) and wild-type axons (B) in worms feeding on E. coli HT115 bacteria grown to different optical density. The underlying numerical data and statistical analysis for each figure panel can be found in S1 and S2 Datasets, respectively. (TIFF) [file pbio.3000638.s001.tiff]

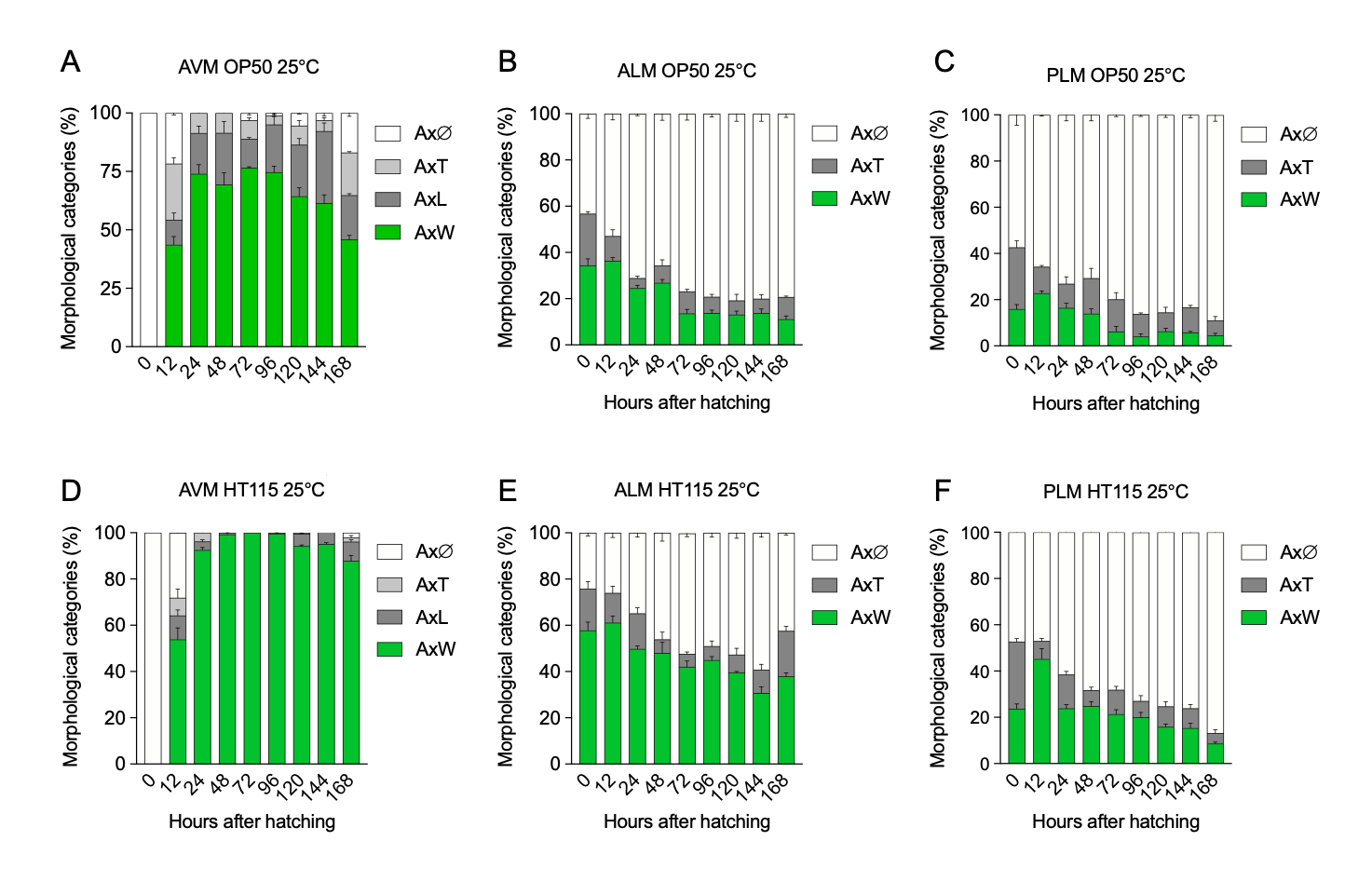

Supplement: S2 Fig — All axonal categories of animals feeding on E. coli OP50 (A, C, and E) and HT115 (B, D, and F) at 25 °C. The underlying numerical data and statistical analysis for each figure panel can be found in S1 and S2 Datasets, respectively. (TIFF) [file pbio.3000638.s002.tiff]

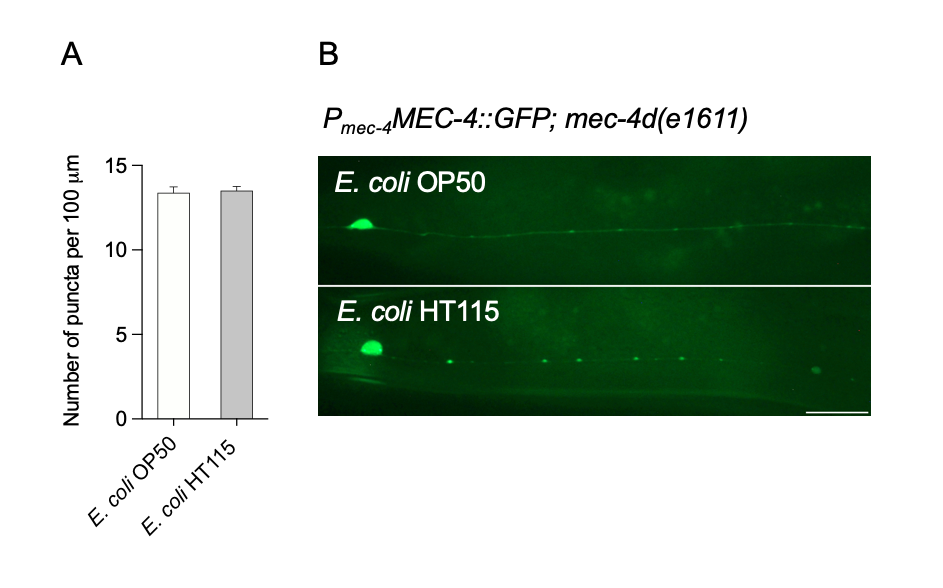

Supplement: S3 Fig — Number of puncta in 100 μm of PLM axons on each bacterial diet (A) and representative photograph of PLM axons used for quantification. Size bar is 20 μm. The underlying numerical data and statistical analysis for each figure panel can be found in S1 and S2 Datasets, respectively. MEC-4, mechanosensory ion channel subunit; PLM, posterior lateral microtubule. (TIFF) [file pbio.3000638.s003.tiff]

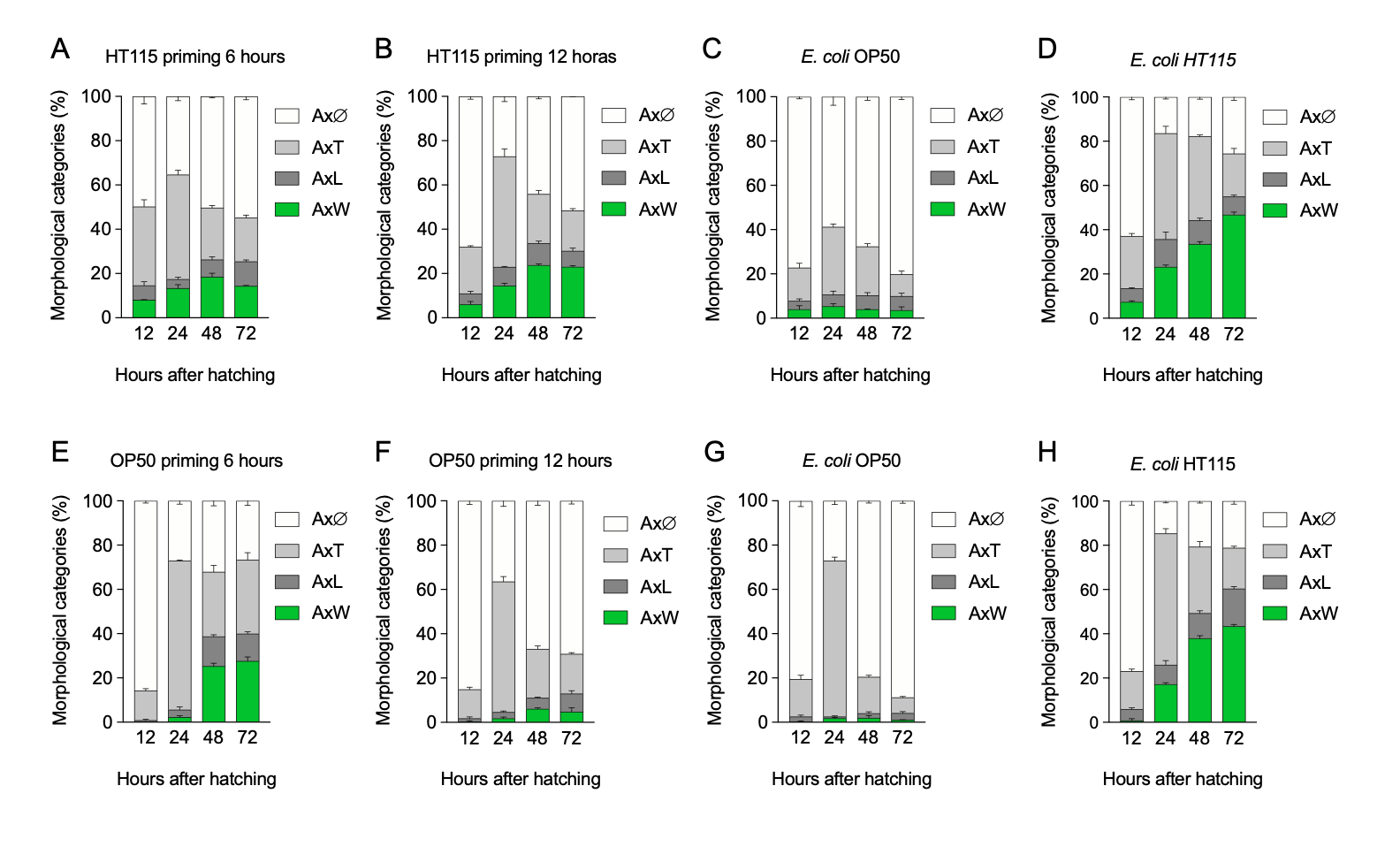

Supplement: S4 Fig — (A–D) All axonal categories of animals feeding E. coli HT115 for 6 (A) and 12 (B) hours with controls of ad libitum E. coli OP50 (C) and HT115 (D) or feeding E. coli OP50 for 6 (E) and 12 (F) hours with controls of ad libitum E. coli OP50 (G) and HT115 (H). The underlying numerical data and statistical analysis for each figure panel can be found in S1 and S2 Datasets, respectively. (TIFF) [file pbio.3000638.s004.tiff]

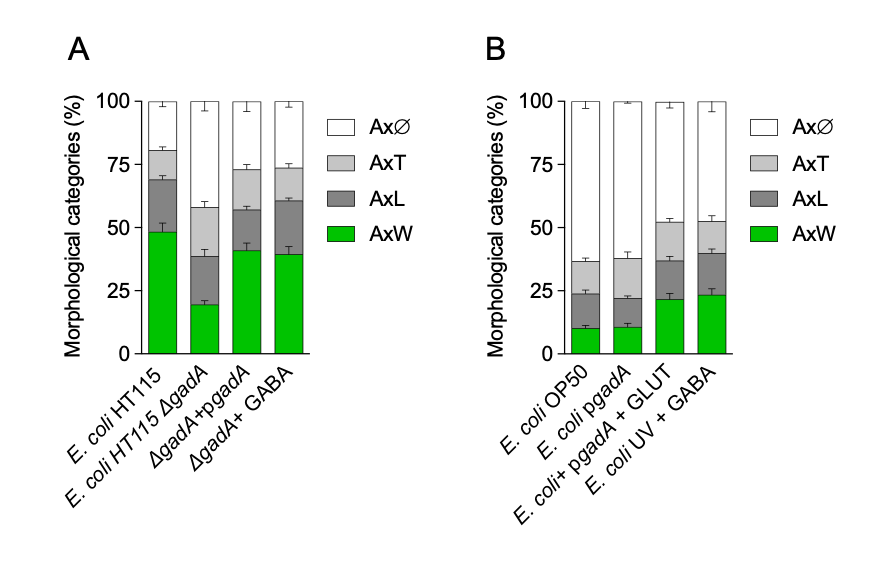

Supplement: S5 Fig — (A and B) All axonal categories of animals feeding wild-type and Δgad E. coli HT115 (A) and OP50 (B) modified with Gad-expressing plasmids, glutamate, and GABA. The underlying numerical data and statistical analysis for each figure panel can be found in S1 and S2 Datasets, respectively. GABA, γ-aminobutyric acid; gad, glutamate decarboxylase enzyme gene. (TIFF) [file pbio.3000638.s005.tiff]

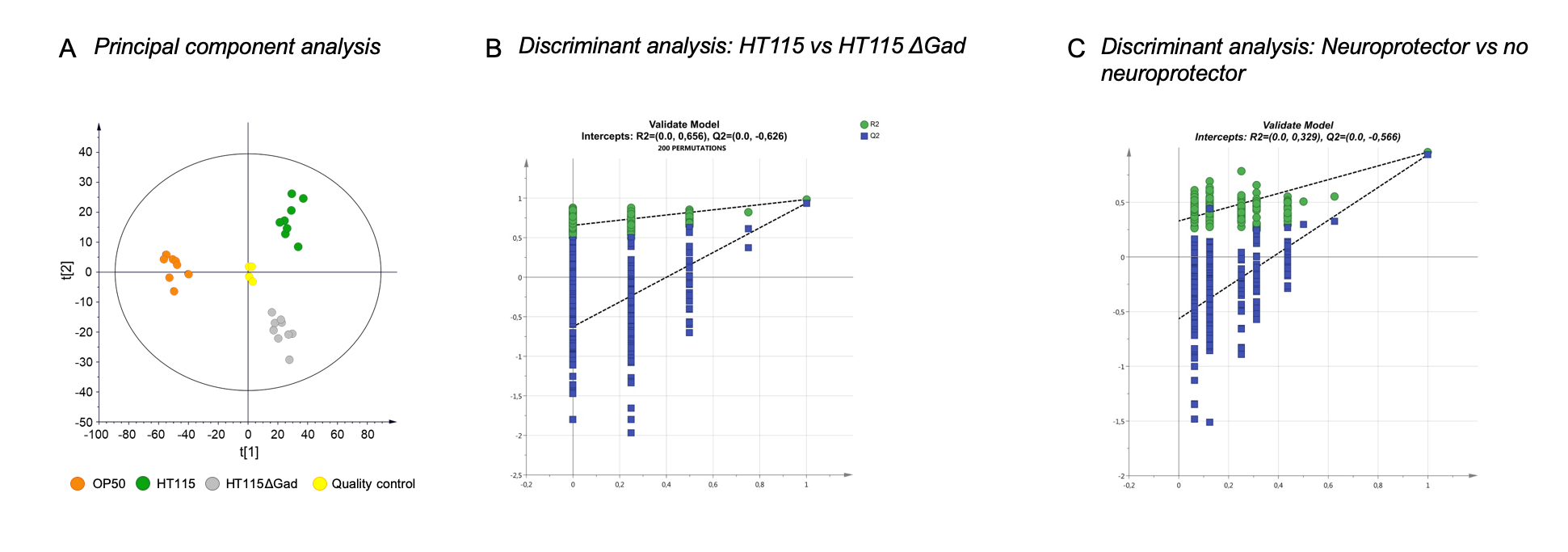

Supplement: S6 Fig — (A) PC score plot derived from 1H NMR spectra indicating metabolic differences between wild-type E. coli strains OP50 (orange) and HT115 (green) and HT115 Δgad mutant (light gray). Quality controls are displayed in yellow. Model parameters are R2X = 0.787 and Q2 = 0.705. (B and C) OPLS-DA validation by 200 permutations. E. coli HT115 and HT115 Δgad validate model intercepts: R2 = (0.0; 0.656) and Q2 = (0.0; −0.626) (B). Protective E. coli HT115 and nonprotective strains E. coli OP50 and HT115 Δgad validate model intercepts: R2 = (0.0; 0.329) and Q2 = (0.0; −0.566) (C). The underlying numerical data for each figure panel can be found in S1 Dataset. gad, glutamate decarboxylase enzyme gene; NMR, nuclear magnetic resonance; OPLS-DA, orthogonal projections to latent structures discriminant analysis; PC, principal component. (TIFF) [file pbio.3000638.s006.tiff]

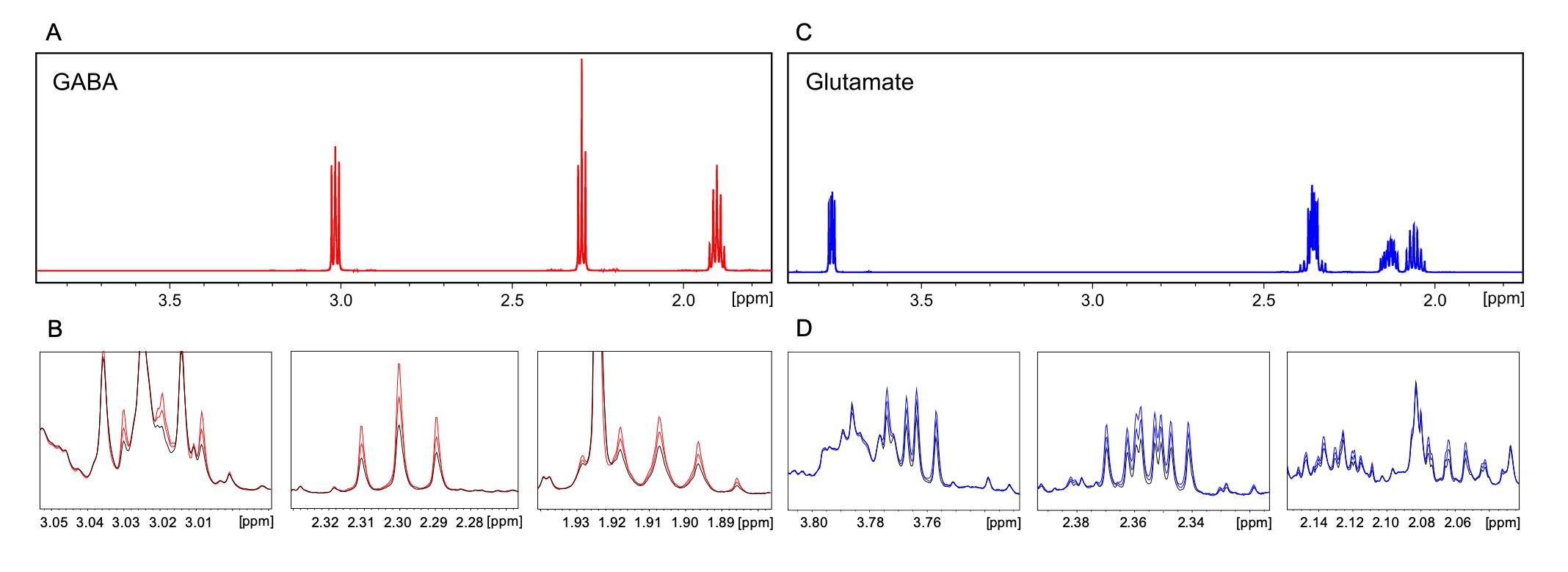

Supplement: S7 Fig — (A and B) 1H NMR spectra of GABA (A) and glutamate (C). (B–D) Spike-in of GABA and glutamate confirms identity of metabolites. E. coli HT115 extract (red) (B); E. coli HT115 Δgad extract (blue). Spike-in was made adding 5 μL of standard 10 mM twice. The underlying numerical data and statistical analysis for each figure panel can be found in S1 and S2 Tables. GABA, γ-aminobutyric acid; gad, glutamate decarboxylase enzyme gene; NMR, nuclear magnetic resonance. (TIFF) [file pbio.3000638.s007.tiff]

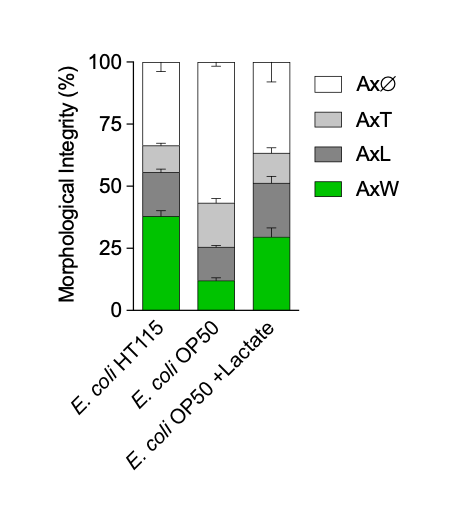

Supplement: S8 Fig — All axonal categories of mec-4d animals feeding on E. coli OP50 supplemented with lactate. The underlying numerical data and statistical analysis for each figure panel can be found in S1 and S2 Datasets, respectively. mec-4d, mechanosensory abnormality protein 4. (TIFF) [file pbio.3000638.s008.tiff]

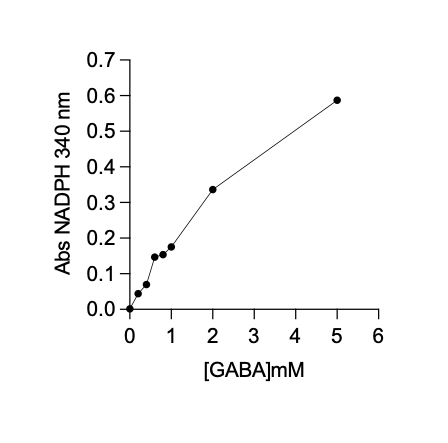

Supplement: S9 Fig — Known GABA concentration plotted against absorbance values creates a curve for later estimation of GABA in samples. The underlying numerical data can be found in S1 Dataset. GABA, γ-aminobutyric acid. (TIFF) [file pbio.3000638.s009.tiff]

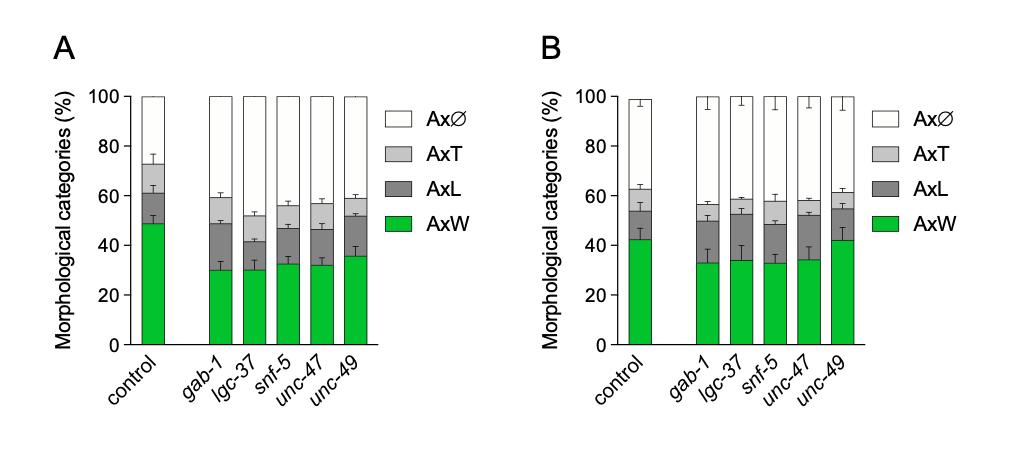

Supplement: S10 Fig — Complete morphological categories of mec-4d animals feeding on E. coli HT115 expressing dsRNA for GABA effector systemically (A) and touch neuron autonomously (B). The underlying numerical data and statistical analysis for each figure panel can be found in S1 and S2 Datasets, respectively. dsRNA, double-stranded RNA; GABA, γ-aminobutyric acid; mec-4d, mechanosensory abnormality protein 4. (TIFF) [file pbio.3000638.s010.tiff]

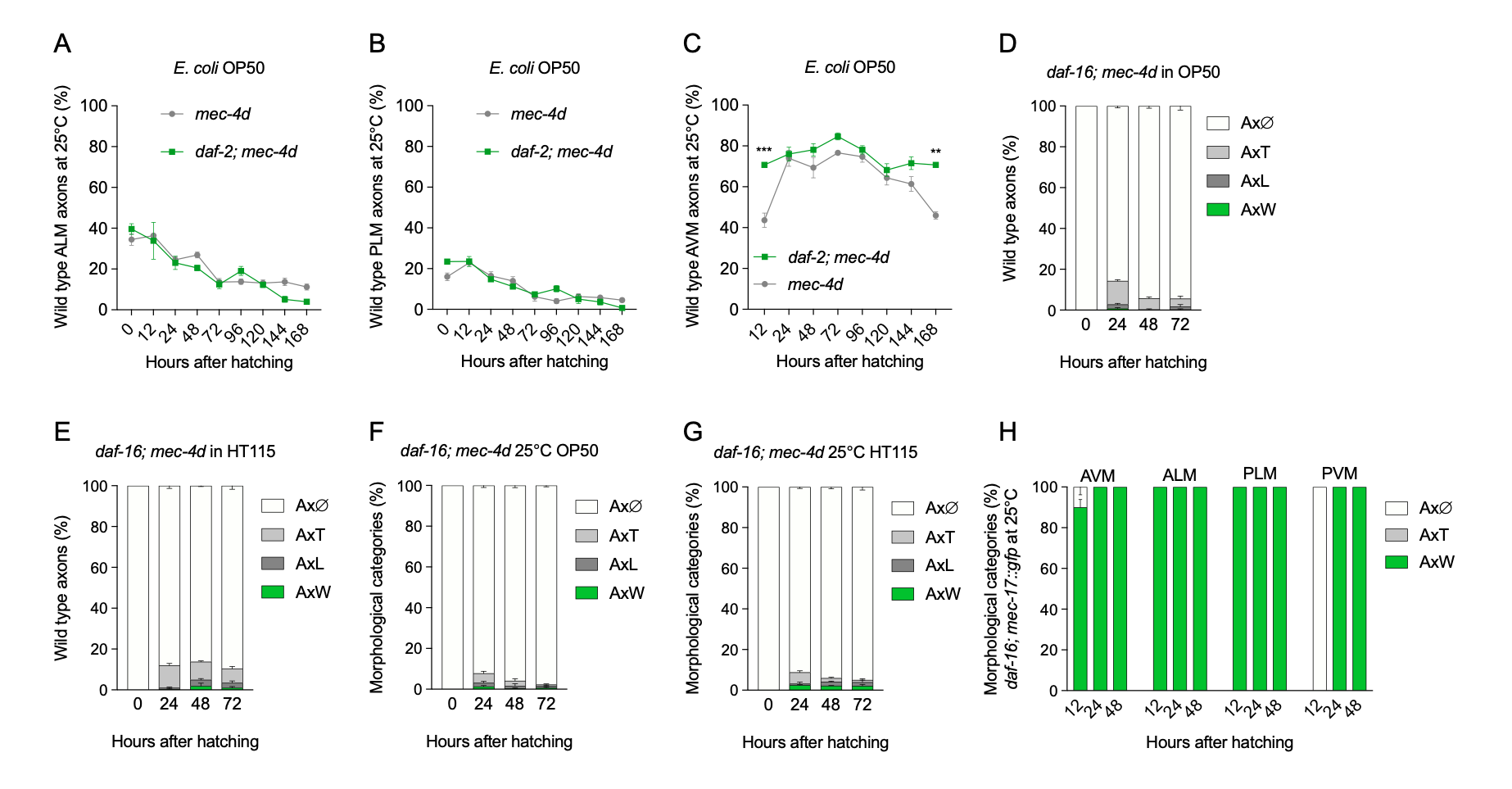

Supplement: S11 Fig — (A–C) Neuronal integrity of AVM (A), ALM (B), and PLM (C) neurons of daf-2(ts); mec-4d animals fed OP50. (D–E) All axonal categories of daf-16; mec-4d animals fed E. coli OP50 (D) and HT115 (E). The underlying numerical data and statistical analysis for each figure panel can be found in S1 and S2 Datasets, respectively. ALM, anterior lateral microtubule; AVM, anterior ventral microtubule; daf-2, codes for insulin-like growth factor 1 (IGF-1) receptor; daf-16, ortholog of the Forkhead box transcription factor; mec-4d, mechanosensory abnormality protein 4; PLM, posterior lateral microtubule. (TIFF) [file pbio.3000638.s011.tiff]
