# Supplementary material for: Bacterially produced metabolites protect C. elegans neurons from degeneration
Source: PLoS Biol. 2020 Mar 24;18(3):e3000638. doi: 10.1371/journal.pbio.3000638 (PMC7092960; doi:10.1371/journal.pbio.3000638)
Supplement: S3 File — Contains sources of experimental materials, strains, and procedures used in this research. MDAR, Materials Design Analysis Reporting. (DOCX) [file pbio.3000638.s017.docx]

Materials Design Analysis Reporting (MDAR)

Checklist for Authors

The MDAR framework establishes a minimum set of requirements in transparent reporting applicable to studies in the life sciences (see Statement of Task: doi:10.31222/osf.io/9sm4x.). The MDAR checklist is a tool for authors, editors and others seeking to adopt the MDAR framework for transparent reporting in manuscripts and other outputs. Please refer to the MDAR Elaboration Document for additional context for the MDAR framework.

Materials

| **Antibodies** | **Yes (indicate where provided: page no/section/legend)** | **n/a** |
| --- | --- | --- |
| For commercial reagents, provide supplier name, catalogue number and RRID, if available. |  | x |
|  |  |  |
| **Cell materials** | **Yes (indicate where provided: page no/section/legend)** | **n/a** |
| **Cell lines:** Provide species information, strain. Provide accession number in repository **OR** supplier name, catalog number, clone number, **OR** RRID |  | x |
| **Primary cultures:** Provide species, strain, sex of origin, genetic modification status. |  | x |
|  |  |  |
| **Experimental animals** | **Yes (indicate where provided: page no/section/legend)** | **n/a** |
| **Laboratory animals:** Provide species, strain, sex, age, genetic modification status. Provide accession number in repository **OR** supplier name, catalog number, clone number, **OR** RRID | All animals were *Caenorhabditis elegans*. Principal strain supplier was *Caenorhabditis Genetic Center* from University of Minnesota.  Strain: wild type  Accession number: N2 (CGC)  Strain: *daf-16(mu86) I; muIs61* [*daf-16::GFP* + *rol-6(su1006)*].  Accession number: [CF1139](https://cgc.umn.edu/strain/CF1139) (CGC)  Strain: deg-1(u38) X.  Accession number: [TU38](https://cgc.umn.edu/strain/TU38) (CGC)  Strains: *uIs31(mec-17*::*gfp);mec-4d(e1611)X*  Accession number: TU2773.  Supplier name: Columbia University, Martin Chalfie [Lab](http://www.columbia.edu/cu/biology/faculty-data/martin-chalfie/faculty.html).  Available strains in Andrea Calixto [Lab](https://www.wormbase.org/resources/person/WBPerson2335#01--10), and previously published in [Caneo, et al., 2019](https://doi.org/10.1371/journal.pgen.1007863).  Strain: *daf-2 (e1368ts)III; uIs31(mec-17*::*gfp)*; *mec-4d(e1611)X*  Strain name: WCH34.  Strain: *daf-16(m27)*, *uIs31(mec-17*::*gfp)*, *mec-4d (e1611) X*  Strain name: WCH39  Strain: *daf-16(m27)*; *uIs31(mec-17*::*gfp)*  Strain name: WCH40­­ |  |
| **Animal observed in or captured from the field:** Provide species, sex and age where possible |  | x |
| **Model organisms:** Provide Accession number in repository (where relevant) **OR** RRID | *Caenorhabditis elegans* |  |
|  |  |  |
| **Plants and microbes** | **Yes (indicate where provided: page no/section/legend)** | **n/a** |
| **Plants:** provide species and strain, unique accession number if available, and source (including location for collected wild specimens) |  | x |
| **Microbes:** provide species and strain, unique accession number if available, and source | Section: Materials and Methods, Bacterial growth.  Page no: 10  *Escherichia coli* OP50, source [CGC](https://cgc.umn.edu/strain/OP50)  *E. coli* HT115, source [CGC](https://cgc.umn.edu/strain/HT115(DE3))  *E. coli* B were obtain from [CGSC](http://cgsc2.biology.yale.edu/Strain.php?ID=16926).  *P. aeruginosa* PAO1 were order from [CGSC](http://cgsc2.biology.yale.edu/Strain.php?ID=16926)  *E. coli* K-12 (BW25113), *Bacillus megaterium,*  *Comamonas aquatica,* and *Comamonas testosteroni* were a gift from Marian Walhout [Lab](https://walhoutlab.umassmed.edu/).  *E. coli* K12 for RNAi silencing clones were from *C. elegans* RNAi collection ([Ahringer library](https://www.sourcebioscience.com/products/life-sciences-research/clones/rnai-resources/c-elegans-rnai-collection-ahringer/)).  *Pseudochrobactrum sp*, *Stenotrophomonas sp, Bacillus pumilus,* were isolated from intestine of individuals of *Caenorhabditis elegans* found in the wild (Santiago de Chile, Huechuraba, Universidad Mayor campus). Bacterium where identify by 16S marker sequencing.  *E. coli* HT115 Δ*gad*.: was built for this research.  Section Materials and methods, Generation of bacterial *gad* mutant. Page no: 13 |  |
|  |  |  |
| **Human research participants** | **Yes (indicate where provided: page no/section/legend)** | **n/a** |
| Identify authority granting ethics approval (IRB or equivalent committee(s), provide reference number for approval. |  | x |
| Provide statement confirming informed consent obtained from study participants. |  | x |
| Report on age and sex for all study participants. |  | x |

Design

| **Study protocol** | **Yes (indicate where provided: page no/section/legend)** | **n/a** |
| --- | --- | --- |
| For clinical trials, provide the trial registration number **OR** cite DOI in manuscript. |  | x |
|  |  |  |
| **Laboratory protocol** | **Yes (indicate where provided: page no/section/legend)** | **n/a** |
| Provide DOI or other citation details if detailed step-by-step protocols are available. | Page no: 10  Section: Feeding RNAi  [Calixto, et al 2012](https://doi.org/10.1371/journal.pgen.1003141.g009).  Page no: 13  Section: Generation of bacterial *gad* mutant  [Datsenko & Wanner, 2000](https://doi.org/10.1073/pnas.120163297).  Page no: 14  Section: GAD enzymatic activity  [Rice, et al., 1993](https://aem.asm.org/content/aem/59/12/4347.full.pdf).  [Yu, et al., 2011](https://doi.org/10.1016/j.enzmictec.2011.06.007).  Page no: 14  Section: Bacterial GABA quantification by GABase assay  [Ippolito & Piwnica-Worms, 2014](https://doi.org/10.1371/journal.pone.0088667).  Page no: 15  Section: Quality controls  [Dona, et al., 2014](https://doi.org/10.1021/ac5025039). |  |
|  |  |  |
| **Experimental study design (statistics details)** | **Yes (indicate where provided: page no/section/legend)** | **n/a** |
| State whether and how the following have been done**, or** if they were not carried out. |  |  |
| Sample size determination | Page no: 17  Section: Sample size |  |
| Randomisation | Page no: 15  Section: Sample preparation for 1H NMR Spectroscopy |  |
| Blinding | Animals were grown in the specific condition without blinding. Blinding occurred at the moment of evaluation of neuronal integrity. A different member of the lab that the experimenter coded the plates with numbers or letters. Code was not revealed until data analysis was finished. |  |
| Inclusion/exclusion criteria | We had a strict exclusion criteria based on contamination by undesired bacteria or fungi. |  |
|  |  |  |
| Sample definition and in-laboratory replication | **Yes (indicate where provided: page no/section/legend)** | **n/a** |
| State number of times the experiment was replicated in laboratory | Page no: 17  Section: Sample size |  |
| Define whether data describe technical or biological replicates | Page no: 17  Section: Sample size |  |
|  |  |  |
| Ethics | **Yes (indicate where provided: page no/section/legend)** | **n/a** |
| Studies involving human participants: State details of authority granting ethics approval (IRB or equivalent committee(s), provide reference number for approval. |  | x |
| Studies involving experimental animals: State details of authority granting ethics approval (IRB or equivalent committee(s), provide reference number for approval. |  | x |
| Studies involving specimen and field samples: State if relevant permits obtained, provide details of authority approving study; if none were required, explain why. |  | x |
|  |  |  |
| Dual Use Research of Concern (DURC) | **Yes (indicate where provided: page no/section/legend)** | **n/a** |
| If study is subject to dual use research of concern, state the authority granting approval and reference number for the regulatory approval |  | x |

Analysis

| **Attrition** | **Yes (indicate where provided: page no/section/legend)** | **n/a** |
| --- | --- | --- |
| State if sample or data point from the analysis is excluded, and whether the criteria for exclusion were determined and specified in advance. | We never excluded points an experiment. Entire experiments were excluded if they contained contamination with undesired microbes. |  |
|  |  |  |
| **Statistics** | **Yes (indicate where provided: page no/section/legend)** | **n/a** |
| Describe statistical tests used and justify choice of tests. | Page no: 17  Section: Statistical evaluation in Dataset 2. |  |
|  |  |  |
| **Data Availability** | **Yes (indicate where provided: page no/section/legend)** | **n/a** |
| State whether newly created datasets are available, including protocols for access or restriction on access. | All data in the manuscript is in Dataset 1. | x |
| If data are publicly available, provide accession number in repository or DOI or URL. | Bacterium Genomes Assemblies  *Escherichia coli* OP50  GeneBank assembly accession number: [GCA_004355015.1](https://www.ncbi.nlm.nih.gov/assembly/GCF_004355015.1#/st),  *Escherichia coli* HT115  GeneBank assembly accession number:  [GCA_004354945.1](https://www.ncbi.nlm.nih.gov/assembly/GCF_004354945.1/#/def). |  |
| If publicly available data are reused, provide accession number in repository or DOI or URL, where possible. |  | x |
|  |  |  |
| **Code Availability** | **Yes (indicate where provided: page no/section/legend)** | **n/a** |
| For all newly generated code and software essential for replicating the main findings of the study: |  |  |
| State whether the code or software is available. | Yes |  |
| If code is publicly available, provide accession number in repository, or DOI or URL. | Page no: 17  Section: Transcriptomic analysis  Detailed pipeline is available at the following link:  https://mlegue@bitbucket.org/mlegue/ht115_op50.git |  |

Reporting

| **Adherence to community standards** | **Yes (indicate where provided: page no/section/legend)** | **n/a** |
| --- | --- | --- |
| MDAR framework recommends adoption of discipline-specific guidelines, established and endorsed through community initiatives. Journals have their own policy about requiring specific guidelines and recommendations to complement MDAR. |  |  |
| State if relevant guidelines (eg., ICMJE, MIBBI, ARRIVE) have been followed, and whether a checklist (eg., CONSORT, PRISMA, ARRIVE) is provided with the manuscript. | We followed the guidelines for *C. elegans* reporting and nomenclature. |  |
