# Supplementary material for: Bacterially produced metabolites protect C. elegans neurons from degeneration
Source: PLoS Biol. 2020 Mar 24;18(3):e3000638. doi: 10.1371/journal.pbio.3000638 (PMC7092960; doi:10.1371/journal.pbio.3000638)
Supplement: Attachment — Submitted filename: Response to reviewers NeuroFood.docx [file pbio.3000638.s020.docx]

Dear Editor,	Santiago de Chile, December 2^nd^, 2019

We have carefully read all comments and addressed experimentally most concerns and questions to the best of our knowledge and effort. Our responses appear in italics after every comment. We thank the reviewers and the editor, for the suggestions have made of this a stronger manuscript.

For this revised version, we devoted a great amount of time to the resolve the role of bacterial metabolites on DAF-16 nuclear translocation, which was a transversal request of all reviewers. We included experiments that were not requested that included lactate, other metabolite only present in neuroprotective bacteria. We also reanalyzed in great depth the transcriptomic results, including other enzymes and transcriptional regulators of the Gad operon, which is central to our conclusions. Importantly, we changed the title of the manuscript to incorporate one reviewer’s request. We also changed GABA for metabolites to include lactate as well.

All of this is detailed below at greater length.

Sincerely,

Andrea Calixto, Ph.D.

Assistant Professor

CGB, Universidad Mayor

Reviewer remarks:

Reviewer #1, François Leulier: This is a timely paper by Urrutia et al., exploring neurodegeneration in a *C. elegans* model, and the effect of the microbial environment in the observed phenotype. The paper is well written and contains an enormous amount of data, which seem to confirm the involvement of a microbial metabolite to protect host neurons from degeneration.
The main conclusions of the paper are:
•       That E. coli strain HT115 specifically protects from a deleterious phenotype in a worm model of neuronal degeneration;
•       That these effects persist with a UV-killed bacterial culture;
•       That protective effects occur between 6 and 12 hours after hatching;
•       That DAF-16 signaling is involved in the protective effect of E. coli HT115;
•       That E. coli HT115 produces the neurotransmitter GABA, which is identified as the main molecule protecting from neurodegeneration.
Overall the paper is clear and easy to read. The controls are well chosen, and the observed phenotypes seem very clear. This article complements the current state of knowledge about bacterial metabolites impacting neuronal processes in the host and would be most welcome in the field of microbiota research.
I have however a few comments, which in my opinion can be quickly addressed by experimental data or by adding a few sentences in the discussion.

MAJOR COMMENTS:
•       While in Figure 1B, *P. aeruginosa* PAO1, a mild pathogen, seems to confer protective effects similar to those of *E. coli* HT115, the only data we have on this strain is about GAD activity in Figure S5. Other soil strains are studied in Figure 7. In my opinion, if authors unequivocally want to prove the effect of GABA-producing bacteria, at least one the experiences should be performed with live or UV-killed P. aeruginosa PAO1, or if possible, with a ΔGad mutant.

*Response: In the revised version we have included:*

*1) Neuronal integrity experiments with UV-killed P. aeruginosa PAO1. This experiment helps us confirm that the protection observed is independent of a metabolite product of the active communication with the host. P. aeruginosa PAO1 is particularly useful for this because as a pathogen it requires to be alive to promote long term defensive behaviors in the worm (Palominos et al., 2017). Results (Figure 2A) show that dead P. aeruginosa PAO1 still confers neuronal protection. Findings are in Results under “Bacterial components promote neuroprotección”*

*2) Gabase measurements for all bacteria used in this work (Figure 8B). It should be noted however, that definitive evidence for the involvement of GABA in the neuroprotective effect was obtained in E. coli. We cannot discard the possibility that other, species-specific molecular determinants are involved in the neuroprotection conferred by P. aeruginosa* *and other bacteria. The experiments with HT115 ΔGad mutant were included in the original manuscript and is now together with all other bacteria in Figure 8A. GAD measurements for ΔGad are on Figure 6A.*

•       In the same manner, I think that priming with UV-killed *E. coli* HT115, as performed in Figure 4, should be addressed. If the metabolite is secreted and there is no nutritional effect, I assume that priming effect should be the same with live or dead bacteria.

*Response: The assumption is correct. The experiment in Figure 4 was performed with UV-killed bacteria (see Materials and methods under* ***Food changes****). We also included the clarification on the result section.*

•       DAF-16 signaling is identified as a main pathway leading to neuroprotection. Unfortunately, this only comes in one figure. I think that, if documented, interactions between DAF-16 signaling and GABA should at least be addressed in the discussion. Moreover, the paper would gain strength if translocation of DAF-16 was absent in *E. coli* HT115 ΔGad.

*Response: Yes, this is a very important point. Following the reviewer’s suggestion, we performed experiments with DAF-16::GFP animals feeding on HT115 Δgad bacteria and scored GFP nuclear localization using two criteria, a quantitative one (number of positive nuclei) and a qualitative one(overall expression). In these experiments, Gad deletion reduced the translocation of DAF::16 to nuclei under both criteria at 24 hours (Figure 10D and E).*

MINOR COMMENTS:
•       I am no expert in *C. elegans*. For a broad audience, such as that targeted by this journal, I think that a small introduction on *mec-4* and its use as a neurodegeneration model would be extremely useful for the comprehension of the paper.

*Response: Thank you. This has now been done. We included concepts such as the permanent opening of the channel as the cause of the degeneration and also that the mutation is a gain of function.*

•       *C. elegans* is always a particular model for gut microbiota research because it is a bacterivore organism, which feeds on bacteria present on the environment. Authors claim that the effects of *E. coli* HT115 are not nutritional, based on the effect of UV-killed bacteria. In a physiological soil environment, how to separate nutritional from non-nutritional effects? I think this could be addressed in the discussion.

*Response: This is a very interesting point for discussion.*

*Traditionally, effects of diet on physiology of C. elegans experiments have been assessed in a reductionist way, using limited numbers of bacteria. As the reviewer points out, C. elegans and its natural diet is a complex system including variable environmental species. Recently, the natural microbiota of C. elegans* *has been estimated (Dirksen et al., 2016)*. *Owed to this, the number of works assessing the effect of metabolites and/or bacterial species on worm physiological responses is rising. For example, the specific metabolic capacities of each strain, and how each member provides nutrients, together with the way multi-species microbiota model affect the system has been determined this year (Zimmerman, J. ISME J. 2019). Thus, knowledge on each nutriment provided by each dietary strain, as well as how the whole system may modify its production and abundance, is now available. In addition to nutritional molecules, modulatory ones are produced by dietary and microbiota species. Studies in humans, where cohorts of health vs disease state can be separately studied, lists of potential neuroactive compounds produced by microbiota metabolism have been generated. Thus, an integration of the metabolic potential of the natural microbiota complex of C. elegans, with the information of predicted effects in other systems, as the human, can be highly informative into the physiological outcomes mediated by specific molecules, pathways and species. In such approach, particular effects as the described in this study have to be simulated and tested in the background of the natural complex microbiota recently modeled in C. elegans.*

*What we intend is to make a difference between bacteria that colonizes the intestines (usually pathogens such as P. aeruginosa) and their effect depend on being alive in the guts, and those (like HT115) that support growth and measurable phenotypes even when they have been UV-killed. We believe all effects are nutritional in the sense that worms need to feed on bacterial cells (detritus makes them starve). What we are trying to separate is the effect of the metabolite by itself (GABA in this case) from a persistent interaction with the host like the one pathogenic bacterium engage in.*

•       Are the strains used in Figure 1 commonly used for *C. elegans* research? The pertinence of the strains should be explained for non-specialists of *C. elegans*.

*Response: Thanks for the suggestion. We have included a paragraph in Material and Methods explaining the pertinence of the use of these strains.*

•       Authors focus on axon growth and degeneration. I wonder if these effects are simply the result of growth and degeneration of neuronal processes, or if it could involve processes of neurogenesis and neuronal apoptosis. How to distinguish between these situations?

*Response: This is a very important point. We do know that degeneration of the touch receptor neurons (TRN) induced by a mec-4d gain of function mutation is non-apoptotic. This was shown earlier by Chalfie and Wolinsky (1990) with the use of caspase mutants. In that work they demonstrated that mec-4d induced death is ced-3 independent. On the issue of neurogenesis: The AVM is born when animals are approximately 12 hours old after hatching. There is no further neurogenesis of these cells. In C. elegans* *the lineage of cells is fixed and neurogenesis for these types of cells has not been described. In a recent paper by our group (Caneo et al., 2019) we showed that there is no neurogenesis of the AVM but axons are capable of regrowing insofar there is a soma left. This happens most notoriously during diapause.*

*This is the reason why we focus on the growth of AVM that occurs shortly after 12 hours post hatching and it is completed soon after (at 24 hours in a wild type background all AVMs are complete, Calixto et al., 2012). In a mec-4d background cells are born and immediately start to degenerate likely because mec-4 is an early expressed gene. What we think is that touch cells are capable of regenerating when the stress generated by the ionic imbalance is lowered. This happens during diapause (Caneo et al., 2019; Calixto, 2015), when DAF-2 is downregulated and probably also under and appropriate bacterial diet (this work). Thus, in previous works, it has been thoroughly demonstrated that in the mec-4d model, degeneration occurs in a non –apoptotic way, and neuroprotection rather than neurogenesis occurs for this cell lineage.*

**Reviewer #2**: The work by Urrutia and colleagues explores the role of bacteria in a *C. elegans* neurodegeneration model. The authors perform a vast array of functional and physiological assays in both *C. elegans* and bacteria to uncover the role of the bacterial metabolite GABA as a key molecule in regulating neuronal decay. While that finding is not entirely unexpected, this work does a great job at highlighting, beyond reasonable doubt, the key role of GABA in regulating this important phenotype. Importantly, the findings of the distal action of GABA on neuronal function are novel, exciting and have wide-relevance for potential biomedical applications. 

The work presented here supports very well all the conclusions put forward by the authors. Importantly, the experimental approaches used are sound and well described, allowing for full replicability of the results.

Points to be addressed:
1) The title is too broad since it is not known whether this is an evolutionarily conserved phenomenon. It should be clearly stated that these findings were obtained in *C. elegans*.

*Response: We see no problem in changing the title. It has now been changed to “****Bacterially produced metabolites protect C. elegans neurons from degeneration”***

2) The authors should show that GABA levels and GAD enzymatic levels from all bacterial strains tested here and with HT115 at different growth times correlate with the worm phenotypes and perform appropriate statistical analysis to show the power of that correlation.

*Response: For the revised version of the manuscript we performed GAD activity (Figure 8A) and GABA measurements (Figure 8B) for all bacteria used in the work. We performed Pearson’s correlation considering GAD activity, GABA production and the wild type axonal category. Correlation is illustrated in Figure 8C. We really appreciated this suggestion.*

*b) The protocol for GABA quantification requires bacteria to be at a high concentration, because at lower concentrations of bacteria this reaction does not have enough resolution.*

*The calibration curve still works very well for 10E8 CFU/ml of bacteria. For this reason, we assessed the quantification of GABA at this concentration only and could not determine it at various growth times*.

3) The authors should show which tissue(s) are important for the role of daf-16 in regulating the neuronal phenotypes.

*Response: One way we foresee this can be addressed is by doing tissue specific RNAi for daf-16. We generated such strains in the past for the touch neurons (Calixto et al., 2012, Plos Genetics). In that work we showed that neuroprotection conferred by daf-2 downregulation was abolished when daf-16 was silenced by TRN specific RNAi. Those experiments were done in HT11, which is universally used in experimental approaches as the RNAi plasmid- holding strain in laboratory C. elegans research. At that time, we did not report any effect by HT115, but that result shows that TRN daf-16 is required for HT115-conferred protection. This is now included in the Discussion section*

4) The authors should attempt to discuss the connection between GABA and the IIS.

*Response: As we conclude after a number of experiments with the DAF::GFP expressing strain, the connection between GABA and DAF-16 translocation cannot be made outside of the HT115 context. OP50 supplementation with GABA or lactate did not increase the number of GFP positive nuclei. We now discuss that the mechanism by which dietary GABA or lactate induce neuroprotection is not mediated by an increase in DAF-16 translocation. However, there is very strong genetic evidence supporting that DAF-16 is necessary for protection to take place, likely because without DAF-16 cells undergoing stress such as the one induced by mec-4d expression have a very low threshold for neurodegeneration.*

5) It is intriguing why HT115 is rewired to produce GABA more than other E. coli strains. Could the authors comment on this based on their genomic and transcriptomic data? It would be interesting to see what happens to this worm phenotype when cultured in an OP50 strain where ssa is deleted.

*Genes coding for GAD enzymes are conserved in the genome of OP50, nonetheless, this strain lacks* rscB*, encoding a transcriptional factor known to be required for the expression of the gad genes in E. coli (Castanié-Cornet MP et al. 2010). This strongly suggests that OP50 lost its ability to produce GABA, instead of HT115 evolving to overproduce it. GABA production usually helps bacteria to deal with acidic conditions. Notably, growth of an E. coli K12 strain in buffered acidic conditions promotes the lost of several systems employed to grow in unbuffered acidic conditions, including GABA production (He, A. et al. 2017). OP50 derives from E. coli B, and this strain was also found to have lost GABA biosynthesis capacity. Thus, this and other traits may be a resemblance of different selective pressures over the E. coli K12 and B lineages.*

*According to our bacteria transcriptomic analysis, there was no differential expression of any Succinyl Semialdehyde related enzyme (transaminase, reductase or dehydrogenase), neither any subproduct was overproduced according to the metabolomic analysis. Even so, SSA production intervenes with other TCA cycle molecules, which affect bacteria homeostasis, also SSA degradation produces NADPH and NADH that are important as a source of energy molecules for neuroregeneration.*

6) metabolites are not over-expressed. Change to over-abundant.

*Response: Thanks, this has now been corrected.*

7) Please correct E. coli K12 or K 12 to K-12. Also, please clarify that HT115 is a K-12 strain and OP50 is a B strain. Finally, state what is the K-12 and B strain used in this study.

*Response: This has now been corrected in figures and along the text.*

Reviewer #3: In this manuscript, the authors use the degeneration of *C. elegans* touch receptor neurons in *mec-4(gf)* animals as a model to investigate the potential effects of dietary bacteria on necrosis/neurodegeneration during development. They report that some laboratory and environmental bacterial strains exhibit more protective effects against degeneration over others. In particular, among the strains tested, *E. coli* HT115 can significantly prevent or protect touch receptor neurons and others in *mec-4(gf)* and *deg-1* animals when compared to *E. coli* OP50, which is routinely used for maintaining and culturing *C. elegans* under laboratory conditions. They further uncover that bacterially produced GABA appears to be critical for the neuronal protection function of HT115. 

The relationship between gut microbiota and brain function has been a hot topic, and some recent studies highlighted the potential link between gut microbiota and certain neurodegenerative diseases. While the paper does nice job in illustrating the protection effects of HT115 in the *mec-4(gf)* degeneration model, the paper falls short in dissecting the mechanism of this protection as well as addressing the broad interest of this discovery. 

Major points
1)      As mentioned in this manuscript, *mec-4(gf)* caused necrosis of TRNs through Na^+^ entry, Ca^2+^ influx, and ROS imbalance. Does the protection effect of HT115 acts through suppressing any of them? if so, is GABA a mediator for this regulation.  The mec-4(gf) and deg-1 both cause neuronal degeneration through necrosis, and the degeneration is relevant to their channel functions, which is a very special type of neuodegeneration and may not be relevant to the common causes of neurodegeneration, such as ageing, neuronal injury, and neurodegenerative diseases. Therefore, the conclusions of this study may not been applicable to a broad concept of neurodegeneration. TNR neurons display degeneration during ageing, and there are some established neurodegenerative disease models in *C. elegans*. It is worth to confirm the board impact of bacterially produced GABA in ageing or disease related neurodegeneration.

*This is a complex point so we will answer in parts.*

As mentioned in this manuscript, *mec-4(gf)* caused necrosis of TRNs through Na+ entry, Ca^2+^ influx, and ROS imbalance. Does the protection effect of HT115 acts through suppressing any of them?

*Response: This question points at the host molecular mechanism of the HT115-meditated protection and it is a matter of future studies in our laboratory. At present we do not know the answer to this question and foresee several years of work to resolve it. The question on whether HT115 changes Na^+^ and Ca^2+^ dynamics within the cell requires electrophysiological recordings on the TRNs, experiments that we consider to be out of the scope of the present manuscript. Nonetheless, these are pivotal issues in our degeneration model and we are on the process of setting collaborations with groups able to apply these techniques. In the near future we will be studying Ca^2+^ dynamics on protected mec-4d neurons. We have been setting up these experiments using genetically encoded calcium sensors. However, in this paper we aimed at discovering whether bacteria promoted neuroprotection. The mechanism of protection in the worm, beyond daf-16 and GABA effectors, is not studied in this work, whose main focus is on the bacterial contribution.*

if so, is GABA a mediator for this regulation.

*We show by RNAi that Systemic GABA effectors are important players in the regulation of neuroprotection. In these experiments TRN specific RNAi did not abolish the effects of HT115 in the worm.*

The mec-4(gf) and deg-1 both cause neuronal degeneration through necrosis, and the degeneration is relevant to their channel functions, which is a very special type of neurodegeneration and may not be relevant to the common causes of neurodegeneration, such as ageing, neuronal injury, and neurodegenerative diseases. Therefore, the conclusions of this study may not be applicable to a broad concept of neurodegeneration.

*Response: We have previously shown (Calixto et al, 2012) that the mec-4d necrosis shares three important characteristics with Wallerian degeneration in mammals: a) It is calcium dependent, b) Mitochondrial permeability pore blockage slows it down and 3) NMNAT overexpression also decreases it. Additionally, we showed that catalases and superoxide dismutases are important for protection which is a common signature of cellular stress in many degenerative diseases. Additionally, the trigger for this degeneration is similar to excitotoxicity for NMDA hyperactivation in mammals.*

*We are confident that mec-4d is a valuable model for studying neuronal death and whose molecular setting is generalizable enough.*

*As far as neuronal injury by laser axotomy, we have recently published a work (Caneo et al, 2019) where we showed that diapause regenerates both mec-4d and axotomized axons. This, while not direct evidence that they share mechanisms, contribute to validate the relevance of the mec-4d-induced death.*

TNR neurons display degeneration during ageing, and there are some established neurodegenerative disease models in *C. elegans*. It is worth to confirm the board impact of bacterially produced GABA in ageing or disease related neurodegeneration.

*Response:* *TRNs do change morphology during ageing (neurite length mainly) but they do not die. Ageing is such a complex and poorly understood phenomena that we have purposely avoided mixing the two concepts. Ageing as such is out of the reach of this work. The impact of HT115 and GABA on ageing may very well be unrelated and entirely a topic of research in itself. Disease models although valuable to make parallels with mammals, have their own peculiarities because they consist on the overexpression of human proteins such as alpha synnuclein and β amyloid peptides and the degeneration time course is longer and less precise than mec-4d death. I would like to stress out that our work consist on unraveling bacterial metabolites that change the course of neuronal death. Therefore we have gone a great length in pursuing their identification.*

2)      The study identifies the difference between HT115 and OP50 that renders the neuroprotective effects, which turns out to be GABA, but does not fully explore the mechanisms of dietary uptake of GABA in promoting neuronal health. The authors did show that GABA receptors and related transporters were involved somewhere outside of touch receptor neurons, but where possibly could it be? It is necessary to know which tissues/cells are the primary targets of GABA, and this data is also essential in understanding how GABA functions in HT115 related neuronal protection. In this manuscript, all analyses of GABA receptors/transports were down using RNAi, and it should be conformed by loss-of-function mutants of those genes as well as tissue special rescue experiments.

*Although we consider that further in deep characterization of the host pathways modified to confer neuroprotection is out of the scope of this first report on the bacterially provided phenotype, we started to elucidate this. We requested the strains for GABA effectors to the CGC. Strains arrived to Chile only recently. We are on the process of constructing the triple mutants (combination of one effector, mec-4d and gfp marker in the touch cells) strains. One observed withdrawn is the fact that the heterozygote mec-4d/+ also degenerates. We are designing strategies to overcome this in sight of a subsequent study.*

*.*

Most of GABA functions are tightly coupled with its concentrations, but it doesn’t seem to be the case in this study. As shown in Fig. 7, in O.P. 50 the GAD enzyme activity and the ability of producing GABA are nearly 50% of that in HT115, but O.P. 50 has no protect effect. In contrast, in Fig.2, 1/100 (1%) dilution of HT115 in O.P. 50, in which the GABA concentration is likely 50%+1% of HT115’s, have almost 50% of protection effects of 100% HT115.  It is difficult to understand that how 1% additional HT115 GABA in O.P. 50, which is already 50% of HT115 GABA, causes such significant changes of phenotypes. To me, it suggests that something else in HT115 may be more important than GABA for its protection effects.

*Response: Correct, this is an important point. Our experiments showed that GABA is involved in protection, but indicated that other molecules could be also involved. For that very reason, we have now also focused on other metabolites that appeared overabundant. We evaluated lactate, which is also diferentially enriched together with GABA,in the protective bacteriacompared to Δ*gad *mutant and OP50. Lactate was very protective (Figure 7G) demonstrating that GABA is not the only metabolite conferring neuroprotection. Consequently, we have adapted the title of the manuscript.*

The data in Fig. 4 is also difficult to understand, that feeding the animals with HT115 before AVM are born can protect them from degeneration. Does this suggest mec-4(gf) is already expressed and cause the degeneration of the Q lineage before AVM are born? Or HT115 causes a long-lasting change, which protects AVM after they are born. If it is the later case, GABA may not be the reason to cause HT115 protection, as GABA neurons are continually generating GABA but failed to protect AVM with O.P.50 feeding.

*Response: Let us explain better:*

The data in Fig. 4 is also difficult to understand, that feeding the animals with HT115 before AVM are born can protect them from degeneration. Does this suggest mec-4(gf) is already expressed and cause the degeneration of the Q lineage before AVM are born?

*Response: Figure 4 shows that HT115 can rescue neuronal protection when animals have been exposed for 6 hours only with OP50. It also shows that HT115 for only 6 hours is not sufficient to produce equivalent protection to HT115 administered continuously. Feeding on HT115 for the first 12 hours (the time of AVM birth) is required to achieve neuroprotection. The reviewer is right in that HT115 fed right previous to the neuron birth is sufficient to confer protection.*

Or HT115 causes a long-lasting change, which protects AVM after they are born. If it is the later case, GABA may not be the reason to cause HT115 protection, as GABA neurons are continually generating GABA but failed to protect AVM with O.P.50 feeding.

*Response: We do think that HT115 causes a long lasting change involving gene expression changes. Importantly,all results indicate that dietary GABA has a specific neuroprotective effect that cannot be achieved by endogenous host GABA..*

In Fig. 5 it seems DAF-16::GFP only has different in 24 h after hatch when feeding with o.p.50 and h115. Does this mean daf-16 is not required for the protecting effects mentioned in Fig.4 when feeding worms only in the first 12 h after hatch

*Response: Let me clarify because this is a rather important point. What we found is that DAF-16 is necessary for survival of neurons under the stress imposed by MEC-4d. Neurons have a normal lifespan without DAF-16 when there is no pro-degenerative stimulus. However, under a ROS inducing, ionic imbalance situation like the one in mec-4d neurons, DAF-16 is essential. In our experiments, DAF-16-defficient cells die earlier and more dramatically. Therefore it is not possible to rescue them with HT115.*

*Translocation of DAF-16 is a different experimental setting. In this case, DAF-16 is expressed at basal levels and what we are assessing is whether there is further translocation to nuclei. This was repeatedly observed only at 24 hours after hatching. Thus, in this setting, although no DAF-16 mobilization to the nuclei is observed at different time points, the protein is being expressed. This condition is different to a DAF-16 mutant.*

3)      The authors also brought DAF-2-DAF-16 signaling into the equation, but it is not genetically clear in what fashion this insulin-like signaling is involved in dietary GABA’s neuroprotective function. daf-2(lf) cause constitutive activity of daf-16, but daf-2(lf) didn’t protect AVM in O.P. 50 feeding (Fig. 5), which suggests that daf-16 activation is not sufficient to protect AVM from degeneration. If that is case, the DAF-16::GFP expression change in Fig. 5D may not be relevant to the phenotype. For the data shown in Fig. 5E-F, it shows daf-16 is involved in AVM degeneration but not necessary involved in HT115 protection.

*Response: Right, the reviewer has pointed at something important. The problem with DAF-2 downregulation is that the intrinsic neuroprotective phenotype at 25°C (the non permissive temperature for this ts allele) is extremely potent. Therefore it cannot get more protected with the mutation. In that context, we had to compare at 20°C where daf-2 allele is incompletely penetrant. Experiment 10A (ex-5A) shows that daf-2, mec-4 is equal to mec-4d which could mean they work in the same pathway or that daf-2 doesn’t have a contribution.*

*Regarding DAF-16 expression and its relationship with the protective phenotype, the reviewer is right.*

*We cannot really know if it is related to the phenotype. We performed a series of experiments with Δgad and also with GABA and lactate supplementation (Figure 10D). They showedthat GABA alone is not sufficient to promote DAF-16 translocation but Δgad mutation does decrease translocation of DAF-16 at 24 hours.*

4)      HT115 feeding may suppress mec-4(gf) expression to protect the cause of AVM degeneration. It should be straightforward to test this by RT-PCR or in vivo labeling of mec-4. 
*Answer: To answer this question we created a double mutant strain expressing the mutant channel mec-4d and an array containing the promoter of MEC-4 and its coding sequence fused to GFP. We scored the number of puncta (MEC-4d channels) in the touch receptor neurons of L4 stage animals feeding OP50 and HT115. Animals had the same number of channels per axon regardless of the bacterial source of food. This finding discards that the diet represses the expression of the channel (S3 Figure).*

Minor points
1)      It will be good to test whether feeding mec-4(gf) with different bacteria affects the its lifespan.

*It is widely documented that the worm lifespan is deeply affected by the type of bacterial diet. Thus, it is highly possible that the different bacteria tested on this study have specific effects on lifespan. Nonetheless, we prefer to maintain the focus of our work in is neurodegeneration during development and early adulthood. We did not address the impact of bacteria on ageing or other life traits. Because this is a very time-consuming experiment and does not really inform us about bacterial contribution to neuronal death, we decided not to do it.*

2)      Fig. 5D, was the nuclear DAF-16::GFP expression assessed based on whole animals or was it neuron-specific? It would be helpful to indicate it in the Methods or figure legends.

*Response: It is now included in the Methods*

3)      Do endogenous GABA expression levels differ between widl-type animals and mec-4d mutants during larval stages?  There is a very good GABA antibody for worms, and it should easy to be tested

*Response: We did not do this because our data supports that the protective GABA is from a bacterial source.*

4)      OP50 + pGadA + glutamic aid can protect neurons from degeneration, but not quite comparable to HT115 or HT115 + Gad mutation + pGadA.

*Response: Right, this shows that GABA is not the only neuroprotective metabolite from HT115. For this very reason we explored lactate as well since it is also a metabolite expressed in neuroprotective bacteria vs non-protective bacteria (Figure 7C and F). Lactate is also capable of protecting neurons (Figure 7G and S7).*

5)      Fig. 1E, statistical significance?

*Response: We have now included the statistical significance.*
