# Supplementary material for: Bacterially produced metabolites protect C. elegans neurons from degeneration
Source: PLoS Biol. 2020 Mar 24;18(3):e3000638. doi: 10.1371/journal.pbio.3000638 (PMC7092960; doi:10.1371/journal.pbio.3000638)
Supplement: Attachment — Submitted filename: Response to reviewers and editors 3.docx [file pbio.3000638.s021.docx]

Dear editors,

We have now modified the text in two of the three aspects you requested. We have also considered the comments made by Reviewer 2. All our responses are in font Times New Roman while your suggestions are in Arial.

Having discussed reviewer 2’s point 1 with the Academic Editor, we do not think you should necessarily address it experimentally. However, while we agree that many of the originally posted questions and doubts which have not been addressed go beyond the current scope of the manuscript, it would be good if the you could add some few sentences to the Discussion to critically discuss other interpretations of how GABA and lactate production could mediate neuroprotection*.*

*R: This has now been done*

Like the reviewers, the Academic Editor thinks you have shown that GABA production is required for neuroprotection, but the mechanism is less clear. For example, it seems that GABA only acts in the context of bacteria being present; therefore, the story is more complicated than presented. The Academic Editor also thinks that, for example, one likely possibility is that GABA and lactate to a large extent actually act within/on the bacteria to allow them to do whatever they do to the host.

*R: Here we were confused and contacted Dr. Gasque by email to explain the following:*

That phrase (one likely possibility is that GABA and lactate to a large extent actually act within/on the bacteria) we do not fully understand, because GABA protects when is supplemented with dead bacteria. All experiments of supplementation are done using UV-killed bacteria. Therefore, GABA protection is independent of bacteria utilizing it or modifying it. Bacteria always has to be present because otherwise worms starve.

What I want to ask is what do the editors mean by "in the context of bacteria being present".

*Dr. Gasque replied that this explanation is satisfactory and this (above) explanation in a letter to Editors would suffice.*

Another unsolved question is the lack of effect on DAF-16 localization with GABA supplementation. Therefore, we think you should be more critical of your findings and discuss the limitation of your data and alternative interpretations to be addressed in future research.

*R: We have now modified the text to address this point.*

*A last point made by the editor by email was the following:*

Another point: please edit your manuscript to clarify you are not monitoring DAF-16 nuclear expression but DAF-16 nuclear ***localization***.

R: We have now used the term **localization** or **translocation** to refer to experiments involving DAF-16::GFP and reserved the term **expression** when we refer to the mutant strain.

Reviewer 2.

Minor points:
1) I don't fully agree with the discussion provided to support their point related to the low production of GABA in B strains. While the rcs may be lost in the B lineage, GABA is still being detected at low levels in OP50. The authors data shows this - approximatelly 50uM, which is far from being neglectable. Therefore, I don't think the authors can say that OP50 lost its capacity to produce GABA. Also, the authors don't explain why HT115 is a particularly high producer of GABA (approximatelly 8-fold higher) when compared to BW25113 which is another derivative of the parental MG1665 K-12 strain. Is this an unexpected trait that only happened to a very highly modified laboratory strain? How does this compare to the parental "wild-type" K-12 strains MG1655 and W3110. The authors should address this experimentally if possible, or alter their discussion to convey their findings more accurately.

R: We have changed the discussion to acknowledge the reviewers point.

2) There is a misspelling in rcsB (the authors named it rscB and rsc) and the nomenclature for K-12 isn't always consistent (sometimes shown as K12).

R: This has now been corrected.

We truly appreciate the work both editors and reviewers devoted to making our manuscript better.

Sincerely,

Andrea Calixto
